# 

**Published:** 1898-02-12

**Authors:** 


**CADBURY'S COCOA** ABSOLUTELY PURE.

### THE HOSPITAL

**CADBURY'S COCOA** NO DRUGS OR ALKALIES USED.

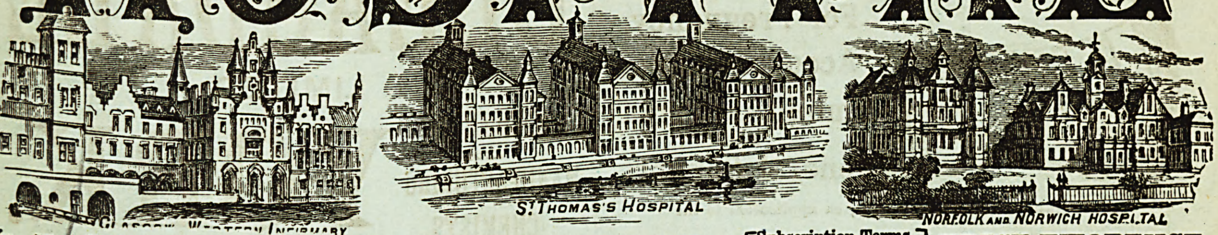

No. 594. Vol. XXIII. [Registered as a Newspaper.] SATURDAY, FEB. 12TH, 1898. [Subscription Terms, see p. 338.] **PRICE TWOPENCE.**

